# ID 331 - Disponibilizaçao de Medicamentos Incorporados: a importância do processo de aquisição para pessoas com restrição ao glúten

**DOI:** 10.5327/2237-9622.2025.v34s1.331

**Published:** 2025-11-25

**Authors:** Luiza Borges Franco, Helaine Capucho, Aline Matilde, Ana Falcomer, Larissa Neves

**Keywords:** glúten, doença celíaca, medicamentos, Farmácia Popular, disponibilização

## Abstract

**Introdução:**

A etapa de disponibilização de tecnologias incorporadas pode ser responsável por riscos à população, caso a aquisição não considere os diferentes excipientes contidos em medicamentos de um mesmo princípio ativo. Entre os riscos, destacam-se aqueles relacionados à presença de glúten, uma proteína que é prejudicial a indivíduos com a doença celíaca (DC), que é uma doença comum na população brasileira. Nesse caso, o único tratamento é a dieta sem glúten, mas mantê-la pode ser difícil devido ao risco de contaminação cruzada ou ingestão acidental por meio de medicamentos, por exemplo.

No Brasil, a Resolução da Diretoria Colegiada (RDC) n.º 768, de 12 de dezembro de 2022, da Agência Nacional de Vigilância Sanitária (Anvisa), exige que as embalagens informem sobre a presença de glúten, açúcar e outros aditivos, a fim de reduzir riscos de contaminação da população que tem restrições a tais componentes. Pelo exposto, o objetivo deste trabalho é identificar a presença de glúten e a comunicação de riscos nos medicamentos para tratamento de hipertensão arterial e de diabetes distribuídos pelo Programa Farmácia Popular (PFP).

**Método:**

Trata-se de estudo transversal, quantitativo, de análise documental. Analisaram-se as bulas de medicamentos registrados no Brasil e que são distribuídos pelo PFP para tratamento de diabetes e hipertensão (lista de fevereiro de 2024). A partir de bula disponível no site da Anvisa, verificaram-se os diferentes excipientes que podem conter glúten. Caso estivesse presente na composição, a bula foi analisada quanto à conformidade com as exigências legais quanto a alertas sobre a presença de glúten no medicamento. Os critérios de inclusão foram medicamentos com registros válidos e com bulas atualizadas e acessíveis no site da Anvisa. Foram excluídos os medicamentos cujas bulas não estavam disponíveis no site da Anvisa.

**Resultados:**

Foram analisados 83 medicamentos para tratamento do diabetes e 212 da hipertensão. No grupo diabetes, 21 medicamentos (25,30%) podem conter glúten e 20 (95,24%) desses medicamentos não incluía na bula a advertência obrigatória. No grupo hipertensão, 142 medicamentos (66,98%) podem conter glúten, mas 99,30% (n=141) deles não possuíam a advertência correta sobre a presença da proteína.

**Conclusão:**

Verifica-se que a população usuária do PFP com diabetes e hipertensão e que tem restrição ao glúten está exposta a riscos por presença da proteína na composição das tecnologias que podem ser adquiridas e distribuídas pelo Programa. Ainda mais preocupante é o fato de que mais de 90% desses produtos não continham a advertência necessária em bula. Considerando que dados da literatura apontam que os fabricantes não dispõem de métodos adequados para garantir a ausência de glúten, bem como não estão preparados para prestar essa informação à população, torna-se evidente a necessidade de melhorar a fiscalização do cumprimento da legislação. Adicionalmente, devem-se educar continuamente os profissionais de saúde acerca desses riscos, já que os estudos apontam para o baixo conhecimento a respeito do assunto. Tais medidas são importantes, mas é essencial que, no processo de disponibilização de tecnologias incorporadas no Sistema Único de Saúde (SUS), possa ser incluída etapa de gestão de riscos para quem tem DC e outras condições de restrição ao glúten, já que pode haver esse risco para tantas outras classes de medicamentos, de forma a proporcionar acesso a um tratamento seguro.

